# Exhaustively characterizing feasible logic models of a signaling network using Answer Set Programming

**DOI:** 10.1093/bioinformatics/btu357

**Published:** 2014-06-24

**Authors:** Carito Guziolowski, Santiago Videla, Federica Eduati, Sven Thiele, Thomas Cokelaer, Anne Siegel, Julio Saez-Rodriguez

Vol. 29, No. 18, 2013, pp. 2320–2326

doi:10.1093/bioinformatics/btt393

The authors would like to note that the joint corresponding authors of *btt393 Exhaustively characterizing feasible logic models of a signaling network using Answer Set Programming* are Drs’ Anne Siegel and Julio Saez-Rodriguez.

